# Birth setting, transfer and maternal sense of control: results from the DELIVER study

**DOI:** 10.1186/1471-2393-14-27

**Published:** 2014-01-17

**Authors:** Caroline C Geerts, Trudy Klomp, Antoine LM Lagro-Janssen, Jos WR Twisk, Jeroen van Dillen, Ank de Jonge

**Affiliations:** 1 Department of Midwifery Science, AVAG and the EMGO Institute for Health and Care Research, VU University Medical Center Amsterdam, Amsterdam, the Netherlands; 2 Department of Women studies, Medical Sciences, Radboud University Nijmegen Medical Center, Nijmegen, the Netherlands; 3 Department of Clinical Epidemiology and Biostatistics, VU University Medical Center Amsterdam, Amsterdam, the Netherlands; 4 Department of Obstetrics and Gynaecology, Radboud University Nijmegen Medical Center, Nijmegen, the Netherlands

## Abstract

**Background:**

In the Netherlands, low risk women receive midwife-led care and can choose to give birth at home or in hospital. There is concern that transfer of care during labour from midwife-led care to an obstetrician-led unit leads to negative birth experiences, in particular among those with planned home birth. In this study we compared sense of control, which is a major attribute of the childbirth experience, for women planning home compared to women planning hospital birth under midwife-led care. In particular, we studied sense of control among women who were transferred to obstetric-led care during labour according to planned place of birth: home versus hospital.

**Methods:**

We used data from the prospective multicentre DELIVER (Data EersteLIjns VERloskunde) cohort-study, conducted in 2009 and 2010 in the Netherlands. Sense of control during labour was assessed 6 weeks after birth, using the short version of the Labour Agentry Scale (LAS-11). A higher LAS-11 score indicates a higher feeling of control. We considered a difference of a minimum of 5.5 points as clinically relevant.

**Results:**

Nulliparous- and parous women who planned a home birth had a 2.6 (95% CI 1.0, 4.3) and a 3.0 (1.6, 4.4) higher LAS score during first stage of labour respectively and during second stage a higher score of 2.8 (0.9, 4.7) and 2.3 (0.6, 4.0), compared with women who planned a hospital birth. Overall, women who were transferred experienced a lower sense of control than women who were not transferred. Parous women who planned a home birth and who were transferred had a 4.3 (0.2, 8.4) higher LAS score in 2nd stage, compared to those who planned a hospital birth and who were transferred.

**Conclusion:**

We found no clinically relevant differences in feelings of control among women who planned a home or hospital birth. Transfer of care during labour lowered feelings of control, but feelings of control were similar for transferred women who planned a home or hospital birth.

As far as their expected sense of control is concerned, low risk women should be encouraged to give birth at the location of their preference.

## Background

The Dutch maternity care system is characterised by the concept that pregnancy and childbirth are basically physiologic processes. Maternity care is divided into midwife-led care, for low risk women and obstetrician-led care for women with an increased risk for complications. Low risk women give birth under supervision of a community midwife and have the choice between home or hospital birth. When complications occur during labour, a woman will have care transferred to an obstetrician-led unit.

Recently it was shown that a substantial proportion of Dutch women look back negatively on their birth experience three years after childbirth [1]. This finding is worrisome and needs further exploration, particularly since childbirth is an important life event that may influence women’s well-being in the short- and long-term [2,3].

The experience of childbirth has several attributes, of which sense of control is a major one [4]. Sense of control is an important predictor of satisfaction with the birth experience [5]. Evaluation of control during labour can be used as a proxy for birth experiences.

Looking back more negatively on the birth experience has been associated with transfer during labour [1]. In the Netherlands the rate of transfer to obstetrician-led care has risen over the last decades. Transfer rates during labour have risen for nulliparous women from 50% in 2008 to 60.3% in 2010 and from 17% to 26.2% for parous women [6,7]. Since transfer of care during labour has become more common, this may affect the birth experience of women.

Recent studies confirm the association between transfer and a negative birth experience, but they did not compare the effect of transfer between a planned home and hospital birth [8,9]. In an older Dutch study, rating of the birth experience after transfer during labour was similar in women planning a home birth compared to women planning a hospital birth [10]. However, currently, it is unknown how the birth experience of women planning a home birth and who are transferred, compares to those planning a hospital birth and who are transferred. Although it has been stated that “the high rate of transfer undercut the raison d’etre of planned home birth” with regard to satisfaction with the birth experience [11], there is no evidence to support this.

To measure sense of control a reliable and valid instrument has been developed: the Labour Agentry Scale (LAS) [12]. Canadian studies using the LAS to compare birth experiences between different birth settings, concluded that planned home birth was related to a higher sense of control during labour compared to planned hospital birth [13,14]. However, in the Netherlands, this has, to our knowledge, never been studied. And, although the Canadian maternity care system shows similarities with the Dutch system, home birth in the Netherlands is much more common (17.1 per cent) [7], compared to Canada (Ontario, 1.6 per cent) [15]. It is important for women to know whether the planned place of birth is associated with sense of control when choosing their birth setting.

Giving birth at home was reported to lead to preserved authority and autonomy whereby the women themselves rule the situation [16]. Women choose home birth to enhance their sense of control over their surroundings. However, the current thought is that positive experiences associated with planned home birth might be overshadowed by negative experiences of women who are moving to hospital if transfer of care to an obstetric-led unit is required [11]. In the Netherlands rates of transfer are relatively high and this might affect the birth experience in the total group of women who plan home birth. Therefore, the hypothesis of this study was that among the total group of women who plan a home birth, whether or not they experienced a transfer, overall feelings of control during labour are lower than women who plan a hospital birth. Women who plan a home birth and who are transferred might be more disappointed if care is transferred to an obstetric-led unit because it means they have to move to hospital and thus not give birth in their chosen setting.

We formulated two research questions (RQ):

1. What is the association of planned place of birth, home or hospital in midwife-led care, with feelings of control during labour experienced by low risk women.

2. What is the association of planned place of birth with feelings of control among women who were in midwife-led care at the onset of labour and who had care transferred to an obstetrician-led unit during labour.

## Methods

In the Netherlands, low risk women receive midwife-led care from community midwives, unless complications arise. For routine antenatal care, a woman visits the midwife in the midwifery practice.

### Study design and study population

The DELIVER study is a multicenter prospective cohort study into the quality, organisation and accessibility of midwifery care in the Netherlands, which was described extensively elsewhere [17].

Briefly, the means of recruitment of clients was through midwifery practices. Purposive sampling was used to select practices, using three stratification criteria: region (north, centre, south), level of urbanisation (urban or rural area), and practice type (dual or group practice). Twenty of the 519 midwifery practices across the Netherlands participated in this study. Between September 2009 and December 2010 client data were collected using questionnaires. Clients who received antenatal care and who gave informed consent were given a brochure by their midwife, with a link to a website where women could fill in up to three questionnaires: one before 34 weeks gestation (the 1st questionnaire), one between 35 weeks gestation and birth (the 2nd questionnaire), one approximately 6 weeks postpartum (the 3rd questionnaire). To improve the overall response, a reminder was sent to all non-responders. In addition, clients who did not complete the questionnaire within one week were called by the research team, and they were invited once more to participate. The response rate of the DELIVER study was 62%.

The DELIVER client data were linked to midwife-led care data from the Netherlands Perinatal Register (PRN, “Landelijke Verloskundige Registratie”, LVR1). Linkage was successful in 86% of the women included in this study. Women with and without linked data were similar with regard to maternal age and ethnic background. Women with LVR1 data linked had a higher socioeconomic status than women without LVR1 data available.

Agreement between LVR1 and DELIVER data for women who started labour in midwife-led care was 99.1% for vacuum or forceps extraction, 99.9% for caesarean section and 99.4 (hospital) to 94.7% (home) for actual place of birth. In case of disagreement, we used data from the DELIVER study.

For this study, participants with singleton term pregnancies that were in midwifery care at the onset of labour were selected. Onset of labour was based on information from LVR1. Women who were transferred for prolonged rupture of membranes (> 24 hrs without contractions) were excluded. Among these women, transfer to secondary care occurred before start of the dilation (first) stage, and thus planned place of birth is unlikely to have affected sense of control. Women who were transferred to secondary care during pregnancy and women who were advised to give birth in hospital in midwife-led care because of a condition that would increase the risk of complications for the woman or baby were also excluded. These conditions are listed in the obstetric indication list (“Verloskundige Indicatielijst; VIL”).

#### Planned place of birth and transfer of care during labour

Planned place of birth (home or hospital under midwife-led care) is recorded on the LVR-1 form at some point during pregnancy.

When complications arise such as listed in the VIL, care is transferred from midwife-led to obstetrician-led care. When a woman is at home, this requires transport to a hospital facility prior to transfer of care, either by car, or in case of an emergency, by ambulance. Transfer of care for women who planned a hospital birth may require transportation from home to hospital in early labour or from a hospital room to another room or another floor within the hospital. However, often no physical transport is necessary, and only the caregiver changes. In this study, both transfer of care during labour or immediately postpartum, were defined as transfer.

#### Sense of control

To measure personal control during childbirth the women filled in a shortened version of the Labour Agentry Scale (LAS), in the postpartum period (on average 6 weeks) on the 3rd questionnaire, twice, concerning feelings of control during the first, the dilatation stage, and the second, the expulsion stage.

The LAS, a self-report scale designed to measure sense of control during childbirth has demonstrated robust psychometric properties with an internal reliability coefficient of 0.97 and evidence of construct validity demonstrated through factor analysis and dual scaling procedures [12]. The LAS has been used in several studies on sense of control in maternity care [13,14,18-20]. The original LAS consists of 29 short affirmative statements (e.g. ‘I felt confident’ and ‘I felt relaxed’). The shorter version of the LAS contains 10 items [21]. We used the LAS-10 to gain insight in feelings of control during both the first and second stage of labour. Translation to Dutch resulted in 11-items, because the English item ‘I felt helpless (powerless)’ was translated into two separate items due to the difference in meaning between ‘helpless’ and ‘powerless’ in the Dutch language. The translated LAS-11 was back-translated into English to check on accuracy of translation. Respondents were asked to rate each statement on a 7-point Likert-scale from (1) ‘never, or almost never’ to (7) ‘almost always’. Coding was reversed on negatively worded items, so that a positive response reflected in a higher score on all items. The separate items were summated to a total score; possible total scores for LAS ranged from 11 (indicating feeling rarely in control) to 77 (reflecting feeling almost always in control). We considered a difference of a minimum of 5.5 points on the 11 item LAS score measured on a 7 points scale as clinically relevant. This is based on studies concerning self-report (quality of life) instruments, which reported that the minimal clinically important difference is half a point on a 7 point scale (0.5 * 11 items is 5.5 points) [22,23].

#### Confounding factors

Maternal age, ethnic background, and social status were taken into account, because of their relation with planned place of birth [24-26] and feeling in control during labour or satisfaction with childbirth [27-30].

For social status we used a score based on postal code, developed by the Netherlands Institute for Social Research (SCP), based on education, income and employment rates, and we linked it to the client data file. A low score equals low social status [31]. Ethnic background was based on the definition of Statistics Netherlands: Dutch (both parents born in the Netherlands), Western background (at least one parent born in another country in Europe except for Turkey, or born in Oceania, Indonesia, North-America or Japan) or non-Western background (at least one parent born in Africa, Latin-America, Asia or Turkey) [32]. This categorisation identifies three separate groups (Dutch, Western and non-Western) based on socioeconomic and cultural aspects.

The birthing process is usually quite different for nulliparous compared to parous women. For parous women the duration of labour is often shorter, they feel more in control during labour [33] and they are far less likely to be transferred to secondary care. We therefore stratified our results for parity.

#### Potential explanatory factors

The effect of transfer (yes/no) on the association between planned place of birth and sense of control was evaluated.

Furthermore, we evaluated the effect of receiving medicinal pain relief (yes/no), because it might be a factor in the causal pathway of the association between planning a hospital birth [34], and sense of control [19]. In addition, anxiety during pregnancy, measured with the Pregnancy Related Anxiety Questionnaire-Revised version (PRAQ-R) score, was assessed as potential explanatory factor, in the relation between planned place of birth and sense of control. Our hypothesis was, that women who are more anxious during pregnancy, both might be more likely to opt for a hospital birth and might be less likely to feel in control during labour. The PRAQ-R score measures anxiety and specific fears related to pregnancy and consists of three subscales [35]. Pregnant women filled in the PRAQ-R in the first questionnaire. The three scales were ‘fear of giving birth’ (3 items for nulliparous women and 2 items for parous women), ‘fear of giving birth to a handicapped child’ (four items) and ‘concern about one’s appearance’ (three items). Items were scored on a four-point scale (4 = very true, 3 = true, 2 = not true, 1 = certainly not true). Higher scores indicated a higher level of anxiety.

The role of medical interventions, including augmentation, vaginal instrumental childbirth and caesarean section were investigated [26,30,36]. Finally, the impact of the baby’s health postpartum on the relation between planned place of birth and feeling in control was evaluated, because that might negatively influence the recall of the birth experience, including sense of control.

### Data-analysis

Baseline and pregnancy related characteristics of low risk women who planned to give birth at home were compared with women who planned to give birth in hospital using mean and standard deviation for continuous variables and numbers with percentages for categorical variables.

For the primary aim of this study (RQ1), the relation between planned place of birth (home/hospital) as independent variable and LAS score of first stage and second stage of labour separately as dependent variables were analysed using multilevel analysis with 2 levels; the midwifery practice level and individual level, to account for clustering of women within midwifery practices. Besides crude analysis, adjustments were made for ethnicity (categorical), maternal age (categorical) and social status (in quartiles). Next, in an additional analysis, possible explanatory factors (i.e. receiving medicinal pain relief, anxiety during pregnancy, transfer, augmentation, mode of birth and complications with baby) were added to the model in addition to the confounders, one at a time. To deal with missing data for anxiety during pregnancy (in the other variables there were only few missings), multiple imputation was performed according to the Predicted Mean Matching method. With this method, each missing value is imputed randomly from a set of nearest observed values in the dataset. Number of imputations was based on the percentage of missing values [37]. Data for PRAQ-R were missing in 31.4% of nulliparous and 23.3% of parous women. All items for anxiety were imputed when missing.

From information on place of birth and transfer of care (extracted from LVR1 forms) we identified women who planned home birth and who were transferred to obstetric-led care during labour or immediately postpartum, (home - transfer); and the women who planned hospital birth in midwife-led care and who were transferred to obstetric-led care during labour or immediately postpartum, (hosp - transfer). With regard to RQ2, we compared the mean LAS score of first and second stage of labour for women who planned a home birth and who were transferred (home-transfer), to women who planned a hospital birth and who were transferred (hosp-transfer). This analysis was adjusted for ethnicity, social status and maternal age.

Furthermore, women who opted for giving birth at home and did (home-home) and women who opted for a hospital birth in midwife-led care and did (hosp-hosp) were identified, as well as women who planned a hospital birth in midwife-led care and who gave birth at home (hosp-home), to gain insight in sense of control among these groups of women in relation to their birth setting using a similar multivariable multilevel model. The group who planned a home birth but actually gave birth in hospital in midwife led care was too small for meaningful analysis (22 nulliparous women and 30 parous women).

For the main analyses we used data from women who started labour in primary care. For some women start of labour in primary care seems likely, but information of the LVR1 data shows discrepancies for the onset of labour. We conducted sensitivity analyses for women with and without discrepancies in the definition for start of labour in primary care.

Since the option for home birth is being questioned with regard to women’s experiences [11], we used hospital birth as the reference group. All analyses were performed using SPSS version 20.0. Statistical significance was considered with a p-value < 0.05.

## Results

**Figure 1:**
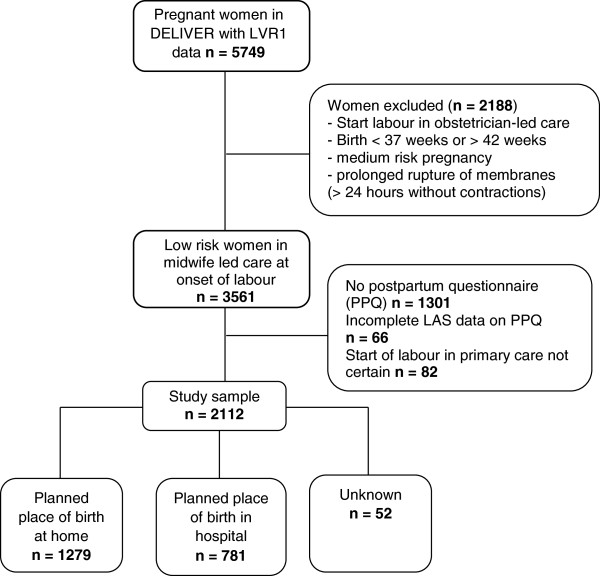
Selection of low risk women who started labour in midwife-led care.

In the DELIVER study, LVR1 data were available of 5749 participants. Of these, 2188 were excluded for medium risk pregnancy, prolonged rupture of membranes without effective contractions, preterm or overdue birth date or start of labour in obstetrician-led care. Of the 3561 remaining women, 3479 started labour in midwife-led care, for 82 women this could not be defined with confidence. The postpartum questionnaire (PPQ) was not filled in by 1301 women and in 66 questionnaires the Labour Agentry Scale was not filled in completely. Of the remaining 2112 eligible women, 1279 women planned a home birth (60.6%) and 781 (36.9%) women planned a hospital birth. Planned place of birth was unknown in 52 women (2.5%) (Figure 1).

The Cronbach’s alpha of the Labour Agentry Scale during first stage and the second stage was 0.85. The mean LAS during first stage and second stage respectively, was 59.6 (SD 12.7)/58.0 (SD 13.9) for nulliparous women and 62.3 (SD 11.4)/59.3 (SD 13.7) for parous women. The ratio nulliparous and parous women was 44:56%. Transfer to secondary care during labour or directly postpartum occurred in 60.6% for nulliparous women and 18,0% for parous women. Main reasons included meconium stained fluid (22%), medicinal pain relief (17%) and failure to progress during first (16%) and second stage of labour (16%).

Table 1 shows that women who choose to give birth in hospital were more likely to be nulliparous, of ethnic minority background and below 25 years or above 35 years, compared to women who choose home birth. Women planning a hospital birth more often had augmentation or were transferred to obstetric-led care and they had a higher rate of instrumental vaginal childbirth and medicinal pain relief. Women in the home birth group were less anxious during pregnancy about giving birth.

**Table 1:** Baseline and pregnancy related characteristics and labour outcomes for planned place of birth of women in the midwife-led care setting at the onset of labour

	**Planned home birth n = 1279**	**Planned hospital birth n = 781**	**Test statistic *X***^***2 ***^**(df)**	**p-value**
**Baseline characteristics**				
Parity, n (%)			11.4 (1)	
Nulliparous	528 (41.3)	382 (48.9)		0.001
Parous	751 (58.7)	399 (51.1)		
Gestational age, n (%)			1.83 (2)	
37 weeks	35 (2.7)	28 (3.6)		0.40
38 – 40 weeks	996 (77.9)	614 (78.6)		
41 - 42 weeks	248 (19.4)	139 (17.8)		
Maternal age, n (%)			8.8 (2)	
< 25 years	99 (7.7)	72 (9.2)		0.01
25-35 years	966 (75.6)	544 (69.7)		
> 35 years	213 (16.7)	165 (21.1)		
Ethnic background, n (%)			54.5 (2)	
Dutch	1160 (90.9)	623 (80.1)		<0.001
Western background	71 (5.6)	74 (9.5)		
Non-western background	45 (3.5)	81 (10.4)		
Social status, n (%)				
1st quartile	342 (26.8)	222 (28.6)	1.1 (3)	0.78
2nd quartile	322 (25.3)	190 (24.5)		
3rd quartile	290 (22.8)	179 (23.1)		
4th quartile	320 (25.1)	184 (23.7)		
**Pregnancy related characteristics**				
Pregnancy related anxiety^^^, median (min – max)				
*Fear of bearing a handicapped child*	8.0 (4–16)	8.0 (4–16)	−1.6*	0.11
*Concern about one’s appearance*	6.0 (3–12)	6.0 (3–12)	−1.3*	0.18
*Fear of giving birth*				
Nulliparous women	6.0 (3–12)	7.0 (3–12)	−3.1*	0.002
Parous women	3.0 (2–8)	4.0 (2–8)	−4.0*	<0.001
**Labour outcomes**				
Medicinal pain relief^†^, n (% yes)	129 (10.1)	171 (22.0)	54.8 (1)	<0.001
Transfer during labour, n (% transferred)	395 (30.9)	362 (46.5)	50.6 (1)	<0.001
Medical interventions, n (%)				
Vacuum-/forceps extraction	111 (8.7)	88 (11.3)	8.1 (2)	0.02
Secondary caesarean section	38 (3.0)	36 (4.6)		
Augmentation, n (% yes)	188 (14.7)	160 (20.5)	11.8 (1)	0.001
Complications baby postpartum, n (%)	21 (1.6)	14 (1.8)	0.067 (1)	0.80

^ This item was missing in 31.4% of nulliparous and 23.3% of parous women.

† Medicinal pain relief includes epidural (172), remiphentanyl (90) and opioids (85).

* z statistic of U (Mann Whitney U test).

### All women

Table 2 shows that planning a home birth is associated with a higher mean score of sense of control, for nulliparous and parous women, during both the first and second stage of labour, taking account of clustering of women within each midwifery practice. Adjusting the multilevel model for ethnicity, social status and maternal age did not influence the association.

**Table 2:** Association between planned place of birth and sense of control (LAS) among women in midwife-led care at start of labour (RQ1)

			**Nulliparous women**		**Parous women**	
LAS* 1st stage						
	**N**	Estimated mean LAS	Difference (95% CI)	**N**	Estimated mean LAS	Difference (95% CI)
**Crude**						
Home	520	60.7	2.8 (1.0, 4.5)**	736	63.5	3.5 (2.1, 4.9)**
Hospital	370	57.9	-	390	60.0	-
**Adjusted**						
Home	515	60.6	2.6 (0.9, 4.3)**	732	63.3	3.0 (1.6, 4.4)**
Hospital	365	58.0	-	386	60.3	-
LAS* 2nd stage						
	**N**					
**Crude**						
Home	500	59.3	3.1 (1.2, 5.1)**	726	60.3	2.8 (1.1, 4.5)**
Hospital	351	56.2	-	386	57.5	-
**Adjusted**						
Home	495	59.1	2.8 (0.9, 4.7)**	722	60.1	2.3 (0.6, 4.0)**
Hospital	346	56.3	-	382	57.8	-

* Labour Agentry Scale (LAS): measured with 11 items on a 7-point Likert scale (min. score 11, max. score = 77).

** p < 0.01.

Crude: multilevel analysis with 2 levels (midwifery practice and pregnant women).

Adjusted: for maternal age, social status, ethnicity (Dutch, western background, non-western background).

The explanatory analysis showed that for nulliparous women, the association between planned place of birth and sense of control during the first stage of labour was partly explained by medicinal pain relief: after adjustment the difference was 1.5 (95% CI −0.2, 3.2). Additional adjustment for, separately, transfer during labour, anxiety during pregnancy, medical interventions (e.g. vaginal instrumental childbirth, caesarean section and augmentation) or neonatal complications within one hour postpartum did not have an effect on the association. In multiparous women, separate adjustment for the abovementioned factors did not change the associations (not shown).

### Women who were transferred

Table 3 (transfer) shows that parous women who planned a home birth and who were transferred to secondary care had higher feelings of control during the second stage of labour compared to parous women who planned a hospital birth and who were transferred. Among nulliparous women who were transferred, feelings of control during second stage of labour were similar for both women who planned a home birth or a hospital birth after adjustment for maternal age, ethnic background and socioeconomic status. During first stage of labour feelings of control among women who were transferred were similar, regardless whether they planned a home or hospital birth.

**Table 3:** Planned place of birth in relation to sense of control (LAS) among women in midwife-led care at start of labour and who were transferred to obstetric-led care during labour (RQ2)

**Transfer**						
			**Nulliparous women**		**Parous women**	
LAS* 1st stage						
	**N**	Estimated Mean LAS	Difference (95% CI)	**N**	Estimated Mean LAS	Difference (95% CI)
**Crude**						
Home-transfer	294	58.6	2.2 (−0.1, 4.5)	95	59.7	3.1 (−0.4, 6.5)
Hosp-transfer	244	56.4	-	108	56.6	-
**Adjusted**						
Home-transfer	292	58.4	1.7 (−0.6, 4.0)	95	58.9	1.8 (−1.8, 5.4)
Hosp-transfer	241	56.7	-	106	57.1	-
LAS* 2nd stage						
	**N**					
**Crude**						
Home-transfer	275	57.1	2.6 (0.1, 5.2)**	91	59.0	5.1 (1.2, 9.0)**
Hosp-transfer	226	54.5	-	104	53.9	-
**Adjusted**						
Home-transfer	273	57.0	2.5 (−0.1, 5.1)	91	58.5	4.3 (0.2, 8.4)**
Hosp-transfer	223	54.5	-	102	54.2	-

* Labour Agentry Scale (LAS): measured with 11 items on a 7-point Likert scale (min. score 11, max. score = 77).

** p < 0.05.

Crude: multilevel analysis with 2 levels (midwifery practice and pregnant women).

Adjusted: for maternal age, social status, ethnicity (Dutch, western background, non-western background).

Home-transfer: women who planned a home birth and who had care transferred during labour.

Hosp-transfer: women who planned a hospital birth and who had care transferred during labour.

Overall, feelings of control for women who were transferred were lower than feelings of control in women who were not transferred (difference in LAS-11 score in 1st stage of labour was 5.3; 95% CI 4.3 - 6.4 and 2nd stage of labour 4.3; 3.1 - 5.6).

### Women who were not transferred

Table 4 (no transfer) shows that women who planned a home birth and who actually had a home birth had statistically significantly higher feelings of control compared to women who planned a hospital birth and who actually gave birth in the hospital in midwife-led care, except for nulliparous women during second stage of labour: no differences in LAS score were observed. Parous women who planned a hospital birth under midwife-led care and who actually gave birth at home under midwife-led care had a statistically significant higher LAS score during second stage of labour, than women who planned a hospital birth and who actually gave birth in hospital under midwife-led care.

**Table 4:** Planned place of birth in relation to sense of control (LAS) among women in midwife-led care at start of labour and who were not transferred during labour

**No transfer**						
			**Nulliparous women**		**Parous women**	
LAS* 1st stage						
	**N**	Estimated Mean LAS	Difference (95% CI)	**N**	Estimated Mean LAS	Difference (95% CI)
**Crude**						
Home-home	204	63.8	3.4 (0.6, 6.2)**	610	64.2	3.6 (1.8, 5.4)**
Hosp-hosp	89	60.4	-	184	60.6	-
Hosp-home	34	63.2	2.9 (−1.6, 7.3)	98	62.8	2.2 (−0.4, 4.8)
**Adjusted**						
Home-home	202	63.6	3.0 (0.2, 5.9)**	606	64.9	3.3 (1.5, 5.1)**
Hosp-hosp	88	60.6	-	182	60.8	-
Hosp-home	33	62.7	2.1 (−2.3, 6.5)	98	62.9	2.1 (−0.6, 4.7)
LAS* 2nd stage						
**Crude**						
Home-home	203	62.2	3.9 (0.8, 7.0)**	605	60.6	3.3 (1.1, 5.5)**
Hosp-hosp	87	58.3	-	184	57.3	-
Hosp-home	35	61.8	3.5 (−1.4, 8.3)	98	61.5	4.1 (0.8, 7.4)**
**Adjusted**						
Home-home	201	61.9	3.1 (−0.1, 6.3)	601	60.5	2.9 (0.7, 5.2)**
Hosp-hosp	86	58.8	-	182	57.6	-
Hosp-home	34	61.6	2.8 (−2.2, 7.7)	98	61.5	4.0 (0.7, 7.2)**

* Labour Agentry Scale (LAS): measured with 11 items on a 7-point Likert scale (min. score 11, max. score = 77).

** p < 0.05.

Crude: multilevel analysis with 2 levels (midwifery practice and pregnant women).

Adjusted: for maternal age, social status, ethnicity (Dutch, western background, non-western background).

Home-home: women who planned birth at home and actually gave birth at home.

Hosp-hosp: women who planned a hospital birth and actually gave birth in the hospital under midwife-led care.

Hosp-home: women who planned hospital birth in midwife-led care and actually gave birth at home.

### Sensitivity analysis

Sensitivity analysis for sense of control and planned place of birth of the 2112 eligible women plus 82 women of which start of labour was unsure, yielded similar results (data not shown).

## Discussion

In this study we showed that women in midwife-led care at the start of labour who planned a home birth with their midwife experienced a higher mean sense of control during labour, than women who planned a hospital birth. Among women who were transferred during labour, sense of control was similar for both women who planned a hospital birth or a home birth. In the second stage of labour sense of control was higher for parous women who planned a home birth and who were transferred, compared to women who planned a hospital birth and who were transferred.

Our study had some strengths and limitations which need to be addressed. We used data from a prospective cohort study. A randomised controlled trial was shown not to be feasible, because women do not accept randomisation for place of birth [38]. Therefore, in this study we have controlled the analyses for confounders to deal with unequally distributed characteristics. Furthermore, in the analysis, we accounted for clustering of women within midwifery practices. For few women planned place of birth was unknown. Some do not choose their place of birth until they are in labour. In some cases the midwife might have forgotten to fill it in. LAS score was not available in all eligible women. It seems, however, unlikely that among non-responders the association between planned place of birth and sense of control would be in the opposite direction. The LAS was filled in an average of 6 weeks postpartum and this raises the possibility of recall bias. However, adjustment for neonatal complications postpartum did not change the results. Furthermore, it has been reported that the LAS remains stable until 3 months postpartum [12]. In the DELIVER study, more participants were highly educated compared to the national female population and between 15 and 45 years of age in 2010 and fewer participants were of non-Dutch origin [17]. With regard to the proportion of nulliparous and parous women and transfer rates, the results in our study were comparable to national rates from 2010 apart from the transfer rate of 18.0% for parous women which was lower than the national rate of 2010 (26.2%) [7]. Women planning a home birth were slightly overrepresented, 60.6% compared to the national percentage of 54% [6], which may be explained by the higher educational level of the participants [25]. A reliability analysis of the 11-item LAS score in our study revealed a high internal consistency.

A good sense of control during labour, is a major contributing factor to a positive childbirth experience [4,5,39]. We found a significant association between a planned home birth and a higher mean score of sense of control during labour. This association was not explained by differences in social status, ethnicity or maternal age. Results of previous studies are consistent with our findings [13,14]. Hodnett found that women who planned birth at home scored 23.8 points higher on the 29-item LAS. However, their hospital births were obstetrician-led at the start, instead of supervised by a midwife, which was the case in our study. Janssen found a higher LAS score of 11.9 on the 29 item LAS scale, for women with a planned home birth. They did not compare sense of control for women who planned a home or hospital birth and who were transferred during labour.

Although we found a statistically significant difference in LAS score between a planned home and hospital birth in our study, the difference was very small and it might not be clinically relevant. In our study a difference of 5.5 points was considered as a clinical important difference [22,23]. Likewise, the difference that was found by Janssen can be considered not clinically relevant, since the difference did not exceed 14.5 points on the 29 item LAS. Among the women who were transferred in our study, no clinically relevant differences were found either. The sense of control scores of women in second stage of labour in our study was higher (mean 58.6) than the LAS-11 reported in a Dutch study into the influence of birthing positions on sense of control during labour (mean 56.2) [40]. Nevertheless, the difference is small and not clinically relevant (< 5.5 points).

In our longitudinal study, information was available from women concerning their pregnancy as well as information concerning labour, which provided insight in background characteristics and labour factors that might give insight in the association between planned place of birth and sense of control. Among nulliparous women, receiving medicinal pain relief explained the difference in sense of control during first stage of labour between women planning home and hospital birth. This could suggest that medicinal pain relief is in the causal pathway: women who plan a hospital birth more often receive medicinal pain relief (our results) and medicinal pain relief has been associated with a lower sense of control [19]. Women who planned a hospital birth but who gave birth at home, had a LAS-11 score similar to women who planned a home birth and who actually had a home birth. This is interesting and could perhaps mean that expectations were surpassed, resulting in a higher sense of control.

A previous study reported that women who were transferred during labour looked back more negatively on their birth compared to women who were not transferred [1]. In particular, it can be hypothesized that unplanned transfer from home to hospital may lead to a reduced feeling of being in control. Since many women with a planned home birth are transferred during labour, these negative experiences might overshadow the positive experiences of women giving birth at home, resulting in an overall reduced sense of control for women planning a home birth. This was also suggested recently, in a clinical opinion report [11]. However, there was no evidence until so far to support this. Our results are not in agreement with this assumption, and show that the mean score of sense of control among women with a planned home birth was not lower than sense of control in planned hospital births. Moreover, we showed that transfer had a similar impact on feelings of control among women who planned a home or hospital birth. Our hypothesis, that transfer would affect birth experiences of women who plan home birth in particular, could not be confirmed.

In our study, feelings of control were lower among women who were transferred during labour, compared to women who were not. We found that birth setting had no influence on this decline. This is in line with previous findings, showing that women who were transferred from midwife-led care at home to obstetrician-led care in hospital during labour, were as positive about the childbirth experience as women who were transferred within the hospital, although this study did not use the LAS [10]. Possibly no clinically relevant difference was found because all women who give birth in hospital need to travel to hospital during labour at some point. However, it is also possible that discontinuation of care as a consequence of transfer might have contributed to the decrease in feelings of control during labour for both groups [19,20]. Unfortunately this could not be explored further, since no data were available on continuous support during labour. Overall, when complications arise and transfer is necessary, levels of fear during labour may increase, which is related to a decreased sense of control [18]. Women hope for or expect a natural birth and do not expect to be transferred. For many women this is disappointing [41]. In addition, women who are transferred have a higher risk of medical interventions, such as augmentation and vaginal instrumental childbirth, which on their own have been reported to reduce feelings of control [30], although the association may be weak [33]. In our study medical interventions did not explain the difference in feeling in control between a planned home and hospital birth.

Our findings can be used when informing women who are in midwife-led care, about the advantages and disadvantages of different places of birth, so that they can make an informed choice. Many women choose a home birth because of a desire of greater personal autonomy [42]. However, there is a considerable chance that they will be transferred during labour, in particular for nulliparous women. It is important for women to know that there is no clinically significant association between planned place of birth and sense of control, and that, when transfer is necessary, feelings of control might decline, but the choice for birth setting has no influence on this decline. Therefore, as far as their expected sense of control is concerned, they should be encouraged to give birth at the location of their preference.

This study focuses on low risk women. To get a broader view of birth experiences of women, it would be useful for future research to compare sense of control among women who receive obstetrician-led care with women in midwife-led care. In addition, with regard to the decrease in sense of control in case of transfer, a qualitative study may provide more in depth insight in the experiences of these women.

## Conclusion

The difference in sense of control during labour was not clinically relevant for low risk women in midwife-led care who planned a home birth compared to women who planned a hospital birth. In women who had care transferred feelings of control were lower. But feelings of control were similar for women who planned a home versus a hospital birth and who were transferred during labour. Low risk women should be informed that planned home or hospital births are associated with similar levels of feeling in control during labour.

### Details of ethical approval

The design and conduct of the DELIVER study was approved by the Medical Ethics Committee of the VU University Medical Centre Amsterdam (2009/284). All participants were informed about the study and they were asked to participate by their consulting midwife. Inclusion of participants occurred on an opting-out basis.

## Competing interests

The authors declare that they have no competing interest.

## Authors’ contributions

All authors contributed substantially to the design of the study, CC Geerts prepared the manuscript and analyzed the data. T Klomp is project leader of the DELIVER study. A de Jonge is the initiator of this study. All authors critically revised earlier concepts of the paper and gave final approval of the version to be published.

## Pre-publication history

The pre-publication history for this paper can be accessed here:

http://www.biomedcentral.com/1471-2393/14/27/prepub
